# Clinical application study of E3D-assisted design combined with personalized 3D printed guide system in the treatment of pelvic fractures

**DOI:** 10.3389/fsurg.2026.1819163

**Published:** 2026-05-20

**Authors:** Zheng Wang, JianJun Liu, Weidong Zhao, Haohui Guo, Jiang Wu, Kai Feng, Di Chen, Jun Li, Zhirong Chen

**Affiliations:** 1 The Department of Orthopaedic Trauma II, Ningxia Medical University General Hospital, Yinchuan, China; 2 First School of Clinical Medicine, Ningxia Medical University, Yinchuan, China; 3 Department of Orthopedics Ward (3), Ningxia Medical University General Hospital, Yinchuan, China; 4 3D Printing Center, Ningxia Medical University General Hospital, Yinchuan, China

**Keywords:** E3D-assisted design, pelvic fractures, percutaneous screw fixation, personalized 3D printed guide system, surgery

## Abstract

**Objective:**

This study compares the clinical efficacy of E3D-assisted design combined with a personalized 3D-printed guiding system (experimental group) and traditional fluoroscopic percutaneous screw fixation (control group) for treating pelvic fractures, confirming the high accuracy of screw placement using the personalized 3D guiding system.

**Methods:**

This study selected 41 patients with pelvic ring injuries treated with minimally invasive percutaneous screw fixation from October 2022 to October 2025. A retrospective analysis was conducted, dividing the patients into two groups: the E3D-assisted design combined with a personalized 3D-printed guiding system group and the traditional C-arm fluoroscopy group. The statistical indicators included operation time, screw accuracy, number of fluoroscopies, intraoperative blood loss, screw types, injury types, fracture reduction quality, and postoperative functional recovery.

**Results:**

The operation time in the experimental group was shorter (experimental group: 53.05 ± 19.04 min vs. control group: 81.05 ± 24.68 min, *p* < 0.001), and screw placement was more accurate in the experimental group (tip: experimental group 4.63 ± 2.72 mm vs. control group 7.38 ± 5.92 mm, *p* = 0.04; pedicle: experimental group 6.75 ± 3.06 mm vs. control group 10.15 ± 1.32 mm, *p* = 0.03; sacroiliac joint: experimental group 5.79 ± 3.38 mm vs. control group 8.80 ± 5.78 mm, *p* = 0.03). There was no significant difference in fracture reduction quality between the two groups.

**Conclusion:**

Minimally invasive treatment of pelvic fractures using E3D-assisted design combined with a personalized 3D printed guiding system has advantages such as shorter surgery time, higher screw placement accuracy, and improved safety. It reduces the learning curve for clinicians, providing significant clinical application value.

## 1 Introduction

With the rapid development of the transportation industry and the acceleration of urbanization, traffic accidents and falls from heights have become frequent, leading to an increasing incidence of pelvic fractures caused by high-energy trauma (1). According to statistics, pelvic fractures account for about 3% of all body fractures, with 10% being unstable fractures, and the mortality rate ranges from 8% to 37% (2, 3). Moreover, pelvic fractures vary in type, often accompanied by massive blood loss and damage to other organs, making surgical treatment challenging and high-risk. It has always been a difficulty in the field of trauma orthopedics (4, 5). Therefore, early and effective stabilization of the pelvic ring to prevent further bleeding and other associated injuries can significantly promote early functional recovery (6).

Traditional surgical methods require extensive exposure of the deep pelvic structures, leading to long operation times, significant trauma, difficult procedures, and excessive bleeding. There is also a risk of injury to the pelvic nerves and major blood vessels during surgery, and the infection rate is higher. Repeated bending of steel plates increases the risk of plate fracture (7). In recent years, with the continuous development of minimally invasive surgical techniques and surgical assistive devices for pelvic fractures, the indications for minimally invasive surgery have expanded, and surgical techniques have matured (8). Minimally invasive surgery for pelvic fractures is increasingly favored by scholars due to its small trauma, less bleeding, lower skin condition requirements, and suitability for emergency situations. Its success depends on achieving intraoperative closed reduction and good intraoperative imaging monitoring. The application of percutaneous screw placement techniques has greatly reduced the trauma of pelvic fracture surgeries and accelerated postoperative recovery. However, due to the irregular anatomical structure of the pelvis, complex surrounding structures, and proximity to vital organs, blood vessels, and nerves, minimally invasive techniques also carry certain risks. In traditional surgery, X-ray imaging is used to monitor the position and direction of screw insertion, which requires long-term training and rich experience from the surgeon. This often leads to multiple attempts, increasing radiation exposure and operation time (9).

With the development of digital orthopedics, computer-assisted orthopedic surgery (CAOS) has greatly improved the accuracy and convenience of surgeries (10). Technologies such as 3D printing, intraoperative navigation systems, and robotic-assisted surgeries have been applied in orthopedics (11), but due to the high cost of hardware and surgical fees, these methods have not been widely promoted, especially in grassroots medical institutions. E3D digital software (Element 3D) is a computer software system from the engineering field, which has also been introduced into orthopedics. As a powerful AE (After Effects) 3D modeling software, E3D not only allows the creation of 3D elements, scenes, and animations but also provides numerous features such as preset bevels, material presets, and model presets. Preoperatively, E3D can provide intuitive and accurate judgments regarding the type of fracture and displacement, as well as simulate the surgery before the actual operation. During surgery, the preoperative E3D surgical plan is referenced, facilitating smooth execution and helping to avoid foreseeable intraoperative complications, offering important guidance for clinical treatment. Additionally, 3D printing technology is utilized to develop and design personalized guiding systems, which are then applied to clinical surgery. Personalized guide designs have become more common in clinical research, with clear advantages, although there are varying degrees of limitations.

Based on this, the project uses the data from the 3D reconstruction of pelvic fractures via CT, assisted by E3D digital software, to design the surgical plan and simulate the surgical process. The surgery is then performed by referencing the preoperative E3D surgical plan to ensure the quality of pelvic fracture reduction. At the same time, an E3D-assisted design combined with a personalized 3D printed guiding system was developed. Using bony landmarks as references and employing a rigid connection device inserted into the bone, the design of the guiding system is unaffected by skin morphology, significantly reducing deviations caused by the guideplate, positioning pins, and skin deformation. The pelvic fracture model in E3D software is used to reverse-engineer data, which is then used to 3D print the personalized guiding system. Finally, the guiding system is used during surgery to accurately place the screws while verifying the accuracy of the pelvic fracture reduction.

Although 3D printing and computer-assisted techniques have been widely applied in pelvic fracture surgery, the present study introduces several methodological refinements. First, E3D software is used not only for three-dimensional reconstruction but also as an integrated platform for preoperative surgical simulation, screw trajectory planning, and postoperative quantitative evaluation, forming a closed-loop digital workflow. Second, unlike conventional skin-surface-based templates, the guiding system in this study is designed based on rigid bone landmark fixation using positioning pins, which reduces the influence of soft tissue deformation and improves intraoperative stability and accuracy. Third, this study applies a standardized multi-point deviation measurement method based on postoperative E3D reconstruction, enabling a more detailed and reproducible evaluation of screw placement accuracy. Therefore, this study aims to evaluate the clinical effectiveness of this integrated approach and to explore its potential as a practical and accessible solution for improving surgical precision.

## 2 Materials and methods

### 2.1 Study design

This is a retrospective analysis study of pelvic fracture patients who received minimally invasive percutaneous screw treatment from October 2022 to October 2025 at the Department of Orthopaedic Trauma II, Ningxia Medical University General Hospital. All patients who met the predefined inclusion and exclusion criteria during the study period were consecutively enrolled, thereby minimizing potential selection bias.

Due to the retrospective design of this study, the sample size was not determined by *a priori* calculation. Instead, it was based on the total number of eligible patients treated within the study period. Therefore, this study should be considered exploratory in nature.

### 2.2 Ethical approval

Each patient underwent a preoperative oral interview and signed a written informed consent form. Ethical approval for this study was obtained from the Medical Research Ethics Committee of Ningxia Medical University General Hospital（Ethical approval number: KYLL-2025-1867）.

### 2.3 Patient selection

Inclusion Criteria: 1. Tile B or C type pelvic fractures; 2. Patients aged 18 years or older; 3. Patients with stable vital signs; 4. Patients with pelvic fractures that were reduced to a satisfactory degree after closed reduction using the UCRT frame; 5. Patients with acetabular fractures that do not require reduction; 6. Patients with sacroiliac ligament injury, sacroiliac joint dislocation, or longitudinal sacral fractures; 7. Type II and III “crescent-shaped” iliac fractures.

Exclusion Criteria: 1. Open pelvic fractures; 2. Patients with severe comorbidities who cannot tolerate anesthesia or surgery; 3. Stable fractures; 4. Pelvic anterior and posterior ring fractures that do not meet the reduction standards; 5.Fracture of the screw insertion point; 6. S1 vertebral comminuted fractures and some sacral Denis Zone III fractures; 7. Iliac wing comminuted fractures that cannot be reduced;8.Skin lesions or infections at the needle insertion site;9.Severe osteoporosis;10.Severe developmental deformities.

### 2.4 Grouping

The included patients were divided into two groups: the E3D-assisted design combined with a personalized 3D printed guiding system group (experimental group) and the traditional C-arm fluoroscopy group (control group). As this was a retrospective study, group allocation was not randomized. Instead, the assignment was primarily based on the availability of 3D printing resources and the feasibility of completing preoperative E3D-assisted planning within the required surgical timeframe, as well as clinical decision-making by the surgical team. Patients were included in the experimental group when preoperative digital planning and guide fabrication could be successfully completed prior to surgery. Otherwise, patients underwent conventional fluoroscopy-guided screw placement and were assigned to the control group. All eligible patients during the study period were consecutively included to reduce selection bias.

### 2.5 3D printed guiding system design and printing

#### 2.5.1 CT acquisition and reconstruction

All patients underwent pelvic CT scanning using a multi-detector CT system. The scanning parameters included a slice thickness of 0.625 mm, with high-resolution bone algorithm reconstruction. The CT data were exported in DICOM format for subsequent processing.

#### 2.5.2 Software and digital planning

The DICOM data were imported into E3D software (Element 3D, version 2.2.2, Video Copilot Inc., USA) for three-dimensional reconstruction, fracture visualization, and preoperative surgical simulation. Model optimization and surface processing were performed using Geomagic Studio (version 12.0, 3D Systems, USA), and guide design was completed using UG/NX software (version 10.0, Siemens, Germany).

#### 2.5.3 3D printing process

The personalized guiding system was fabricated using a Formlabs Form 3B 3D printer (manufacturer: Formlabs Inc., USA). The printing material used was biocompatible photosensitive resin. After printing, the guide was post-processed, cleaned, and sterilized using low-temperature plasma sterilization prior to surgical use.

#### 2.5.4 Time requirements

The average time required for the complete workflow, including CT data processing, preoperative planning, guide design, 3D printing, and sterilization, was approximately 2–3 days, depending on case complexity and printing queue.

#### 2.5.5 3D printing process

**Figure 1:**
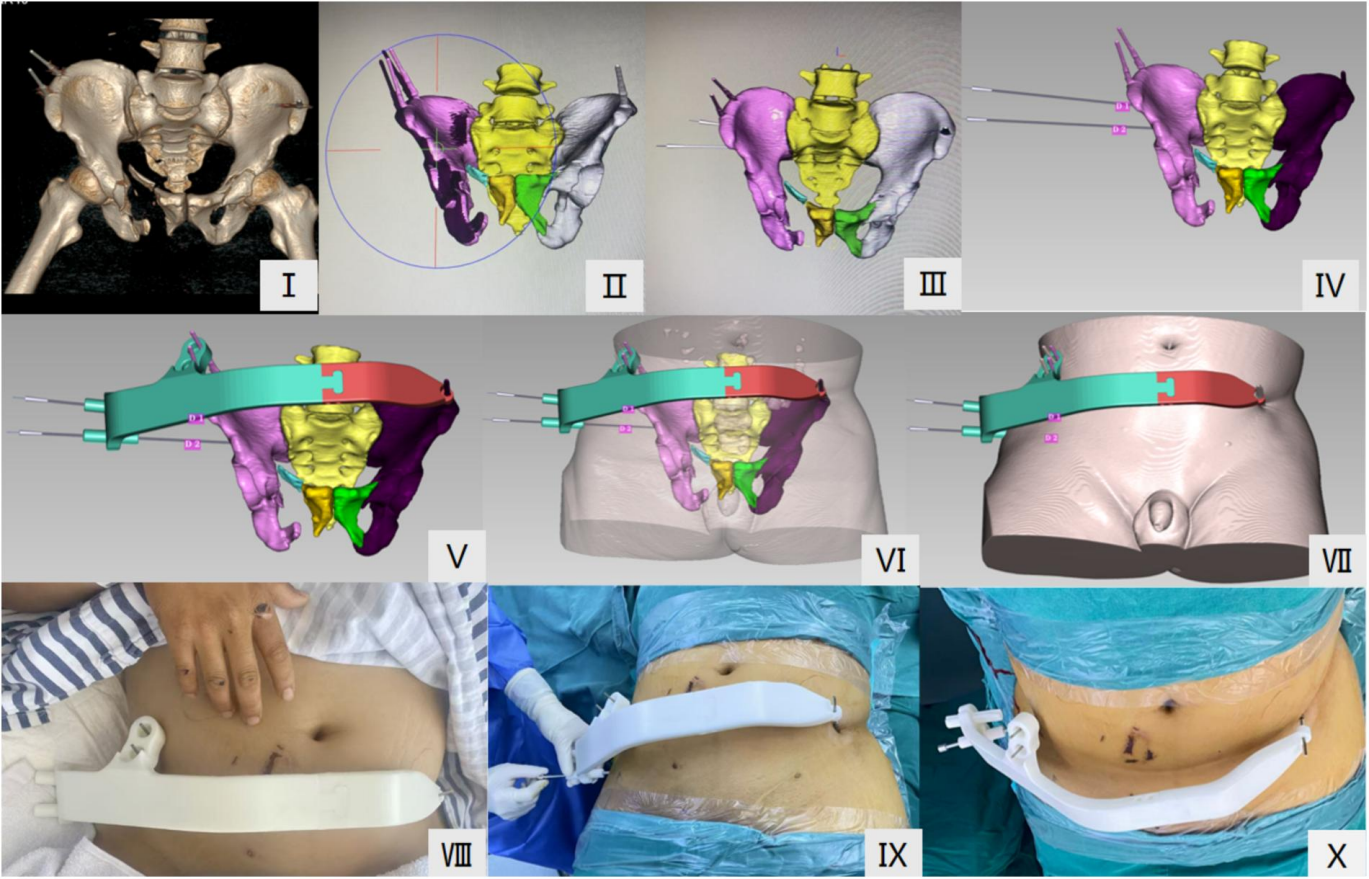
Preoperative design of the E3D-assisted personalized 3D printed guiding system. **(I)** Pelvic CT scan and 3D reconstruction after insertion of the positioning pins. (**II, III, IV**) Surgical simulation for fracture reduction and screw channel design. **(V)** Reverse engineering of the personalized 3D printed guiding system. (**VI, VII**) Construction of skin tissue. (**VIII**) Preoperative assembly. (**IX, X**) Intraoperative assembly and application.

1. After the patient was admitted to the emergency department and the positioning pins were inserted, a pelvic CT scan was performed (Figure 1I). The CT data were then imported into E3D software to generate a pelvic fracture model (Figures 1II–IV), and a simulated surgical reduction was performed on the model. After achieving a satisfactory reduction, the final model was saved as an STL file.
2. The STL format file was imported into Geomagic Studio 12.0 (Unigraphic Solution, USA) image processing software, where the model was repaired and smoothed by removing sharp protrusions and filling in depressions. After processing, an X-t format model was generated. The X-t format model was then imported into UG Geomagic Studio 2012 (Rainidrop, USA) software, where a 3D printed guiding system digital model, matching the pelvic fracture, was generated (Figures 1V–VII).
3. Using 3D printing technology, a personalized 3D printed guiding system was generated through reverse engineering, while simultaneously printing the patient's personalized 3D printed guiding system based on the simulated surgery in E3D software (Figure 1VIII).
4. The personalized 3D printed guiding system was applied to the post-reduction pelvic fracture 3D printed model for screw placement accuracy verification. After satisfactory verification, the system was sterilized using low-temperature plasma technology and properly stored for use during surgery.

This study collaborated with the 3D Digital Orthopaedics Center of Ningxia Medical University General Hospital. The average time from generating the personalized 3D printed guiding system based on the reference pelvic CT to determining the surgery was 2−3 days, including the sterilization process and transportation.

### 2.6 Surgical procedure

Coloclysis was performed on all patients the night before surgery to prevent intraoperative fluoroscopy from being affected by intestinal gas and bowel contents. A fluoroscopic surgical table was used to facilitate intraoperative radiography of all pelvic positions. Each patient was placed in the supine position. Based on the patient's body type, a cushion was stacked to a height of approximately 2–3 cm at the waist to provide space for screw insertion.

Both groups of patients were under general anesthesia and placed in the supine position on a fluoroscopic traction operating table. The upper limbs were abducted and extended, positioned on the arm boards extending from the operating table. A cushion was placed under the lumbar-sacral area, elevating it by 2–3 cm to provide space for screw insertion. For patients with Tile C1 type fractures, traction was applied to the affected side's lower limb, with the traction system connected to the foot of the table. Standard disinfection and sterile draping were performed, followed by the installation of the UCRT pelvic reduction frame. The pelvic fracture was reduced through unlocking, rotation, and traction, with fluoroscopic images taken in three views (anterior-posterior, inlet, and outlet) to confirm the reduction of the pelvic ring.

The experimental group first installed the personalized 3D printed guiding system. If the guiding system could not be installed smoothly, it indicated that the reduction was unsatisfactory; if it was installed smoothly, it confirmed that the reduction met the preoperative plan **(**Figures 1IX,X**)**. Then, according to the preoperative plan, a skin incision approximately 1 cm in length was made, and a guiding sleeve was inserted until it reached the bone surface. The surgeon then used an electric drill to insert a 2.5 mm guiding pin along the direction of the guiding sleeve. After confirming the position and depth of the guiding pin with C-arm fluoroscopy, the guiding system was removed, and the length of the pin within the bone was measured, which was found to be consistent with the preoperative plan. A hollow lag screw (diameter 7.3 mm, Huason Company, China) was then inserted along the pin. After the screw placement, fluoroscopic images were taken in the pelvic anteroposterior, inlet, outlet, obturator oblique, and iliac oblique views to confirm the proper position of the screw and ensure no abnormalities in the hip joint. The wound was irrigated, and the skin was sutured.

**Figure 2:**
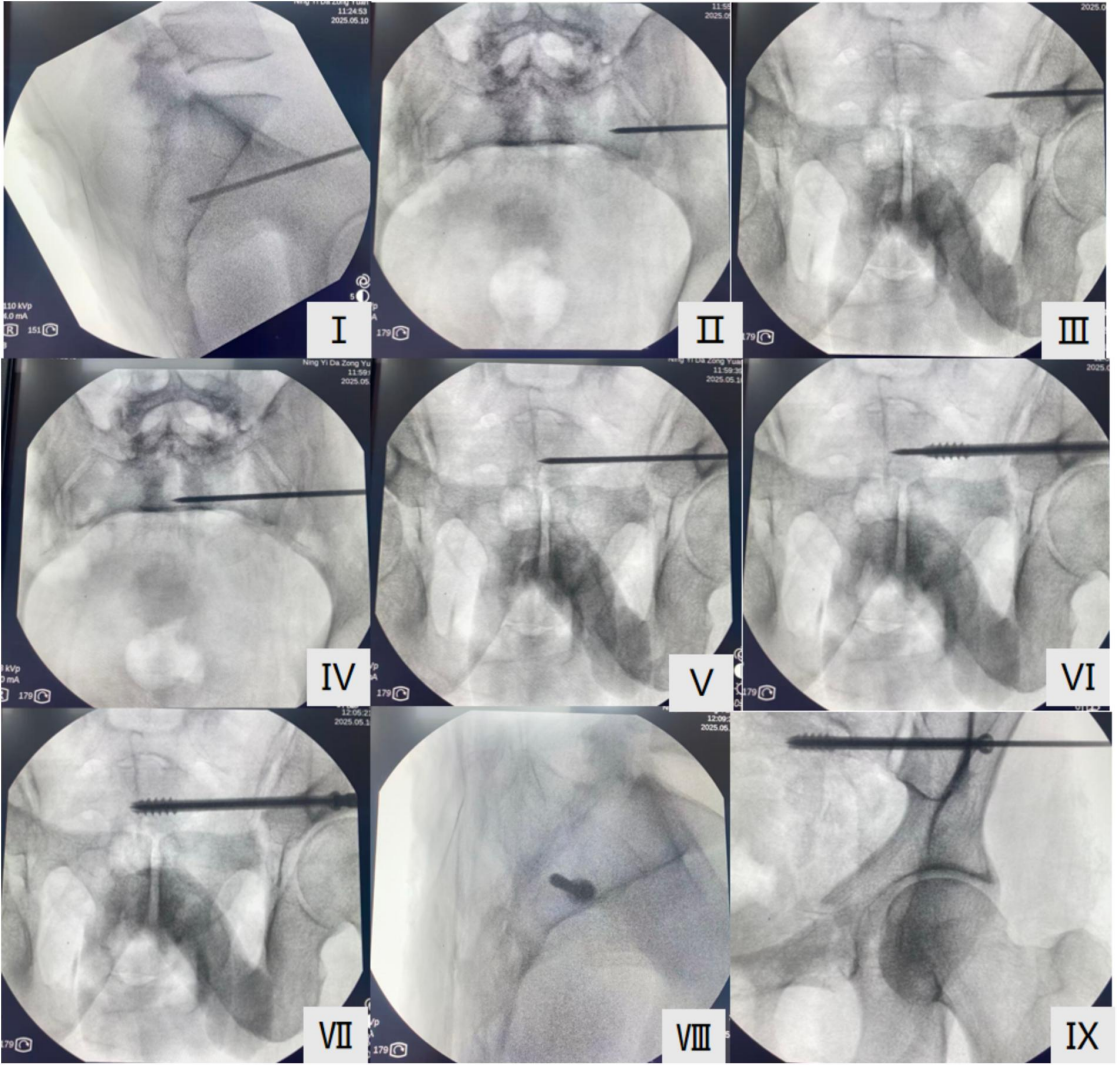
Screw placement process under C-arm guidance. (**I, II, III**) K-wire positioning in pelvic lateral, inlet, and outlet views. (**IV, V**) After confirming the K-wire position, it is placed in the vertebra. (**VI, VII)** Screw placement along the K-wire trajectory. (**VIII, IX**) Verification of screw position on the pelvic lateral and anteroposterior x-rays.

In the control group, traditional freehand screw placement was used under C-arm fluoroscopy guidance, with verification through the pelvic lateral, inlet, and outlet views (Figures 2I–III). The 2.5 mm guiding pin was manually drilled into the bone along the direction of the guiding sleeve (Figures 2IV,V). After confirming the position, a hollow lag screw was inserted (Figures 2VI–IX).

### 2.7 Postoperative management

Within 24 h post-surgery, patients routinely receive cefazolin to prevent infection. Starting from 24 h post-surgery, low-molecular-weight heparin calcium is used to prevent deep vein thrombosis until discharge, after which oral rivaroxaban tablets are administered. Strengthening isometric muscle contraction exercises for both lower limbs, and observing the patient's postoperative functional recovery.

Postoperative rehabilitation: Starting on the first day after surgery, patients perform once-daily extreme passive hip and knee flexion exercises to prevent joint adhesion, ensuring that both the hip and knee flexions exceed 90°. On the second day post-surgery, patients are instructed to sit up and engage in active exercises for both lower limbs without weight-bearing, along with postoperative X-ray and CT re-examination. Postoperative follow-up X-rays are taken at 1, 3, and 6 months to assess fracture healing, and the start of weight-bearing exercises is determined based on the X-ray results.

### 2.8 Outcome measures

Operation time, number of fluoroscopic examinations, and intraoperative blood loss were recorded to evaluate the efficiency of iliosacral screw placement, while screw accuracy was recorded to assess the precision of iliosacral screw insertion. The quality of fracture reduction was evaluated using the Matta criteria (12), based on the maximum residual displacement observed on anteroposterior, inlet, and outlet pelvic radiographs: excellent (≤ 4 mm), good (5–10 mm), fair (11–20 mm), and poor (> 20 mm). Clinical function was assessed using the Majeed scoring system, which includes seven domains: pain, work status, sitting, standing, gait, walking distance, and sexual function. According to the Majeed pelvic functional score (13), recovery outcomes were classified as excellent (> 85 points), good (70–84 points), fair (55–69 points), or poor (< 55 points). Follow-up evaluations were conducted at 1, 3, and 6 months postoperatively in the outpatient clinic or via online follow-up to assess fracture healing and postoperative functional recovery. Screw placement accuracy measurements were independently performed by two experienced orthopedic surgeons who were not involved in the surgical procedures. However, formal intra- and inter-observer reliability analyses were not conducted.

### 2.9 Statistical analysis

All statistical analyses were performed using SPSS software (version 26.0; IBM Corp., Armonk, NY, USA). Continuous variables were expressed as mean ± standard deviation (SD), while categorical variables were presented as frequencies and percentages. The Shapiro–Wilk test was used to assess the normality of continuous data. For normally distributed data, comparisons between the two groups were performed using the independent samples t-test. For non-normally distributed data, the Mann–Whitney U test was applied. Categorical variables were analyzed using the chi-square test or Fisher's exact test, as appropriate. Ordinal variables, such as fracture reduction quality and functional outcomes, were analyzed using the rank-sum test (Mann–Whitney U test). The homogeneity of variances was assessed using Levene's test prior to applying parametric tests. If variance homogeneity was not satisfied, a corrected t-test was used. All statistical tests were two-tailed, and a *p*-value of < 0.05 was considered statistically significant.

## 3 Results

### 3.1 Patient demographics and follow-up

Based on the inclusion and exclusion criteria, a total of 41 patients were selected. Among them, 20 patients were treated with E3D-assisted design combined with a personalized 3D printed guiding system (experimental group), and 21 patients received C-arm fluoroscopy treatment (control group). The average age of the experimental group was 40.20 ± 14.10 years, including 10 males and 10 females. The average age of the control group was 38.68 ± 12.12 years, including 13 males and 8 females (*p* = 0.71). All patients experienced high-energy trauma, including traffic accidents (10 cases in the experimental group, 11 cases in the control group), falls from height (5 cases in the experimental group, 5 cases in the control group), and crush injuries (4 cases in the experimental group, 6 cases in the control group). No significant differences were found in the above data, indicating that comparisons of other data between the two groups are feasible **(**Table 1**)**.

**Table 1:** Data of patients.

Category	Template Group (*n* = 20)	Control Group (*n* = 21)	Test Value	*p* Value
Sex			0.589	0.44
- Male	10	13		
- Female	10	8		
Age (years)	40.20 ± 14.10	38.68 ± 12.12	0.372	0.71
Operation time (min)	53.05 ± 19.04	81.05 ± 24.68	-4.051	<0.001
Number of fluoroscopies	48.60 ± 7.93	54.24 ± 9.32	-2.081	0.04
Blood loss (mL)	16.59 ± 9.22	19.71 ± 6.88	-1.459	0.15
Screw Placement Accuracy				
- The deviation at the tip	4.63 ± 2.72	7.38 ± 4.01	-2.068	0.04
- The deviation at the pedicle	6.75 ± 3.06	10.15 ± 1.32	-2.322	0.03
- The deviation at the sacroiliac joint	5.79 ± 3.38	8.80 ± 5.78	-2.200	0.03
Number of screws				
- S1	11	14		
- S2	14	9		
- Total	25	23		
Mechanism of injury				
- Traffic accident	10	11		
- Crushing	4	6		
- Falling	5	5		
Quality of reduction			1.083	0.58
- Excellent (≤4 mm)	13	12		
- Good (4−10 mm)	7	8		
- Fair (10–20 mm)	0	1		
Functional outcomes			0.531	0.91
- Excellent (≥85)	8	10		
- Good (80-74)	7	7		
- Fair (69-55)	2	1		
- Poor (<55)	3	3		

### 3.2 Operation time

Operation time was measured from the start of anesthesia to the completion of the surgery. The average operation time in the experimental group was 53.05 ± 19.04 min, compared to 81.05 ± 24.68 min in the control group, with a statistically significant difference between the two groups (*p* < 0.001) **(**Table 1**)**.

### 3.3 Intraoperative fluoroscopy count

The number of screws placed was recorded, and the average fluoroscopy count for both groups was calculated. A total of 48 screws were inserted in 41 patients: in the experimental group (20 patients), 25 screws were placed (11 in the S1 segment, 14 in the S2 segment); in the control group (21 patients), 23 screws were placed (14 in the S1 segment, 9 in the S2 segment). The average number of fluoroscopies in the experimental group was 48.60 ± 7.93, while in the control group it was 54.24 ± 9.32. The difference between the two groups was statistically significant (*p* = 0.04) **(**Table 1**)**.

### 3.4 Intraoperative blood loss

The average blood loss during the screw placement process was recorded. The average blood loss in the experimental group was 16.59 ± 9.22 mL, and in the control group, it was 19.71 ± 6.88 mL. It is evident that the blood loss in the control group was higher than that in the experimental group, but the difference between the two groups was not statistically significant (*p* = 0.20) (Table 1).

### 3.5 Screw placement accuracy

Postoperative routine pelvic CT was performed, and the pelvic CT data were imported into E3D software to establish cross-connections of the coronal, sagittal, and axial views. The ideal screw position was considered to be fully within the cortical margin of the sacrum, parallel to the corresponding anterior and posterior sacral endplates. For S1 screws, the ideal position was the midpoint between the S1 superior sacral endplate and the S1 neural foramen, and for S2 screws, it was the midpoint between the S2 superior endplate and the S2 neural foramen. Using E3D software, standard screw channels were constructed, and the ideal screws were pre-set. The deviation between the actual screws and the ideal screws was measured at the tip, sacral pedicle, and sacroiliac joint on the sagittal view. This was done to evaluate the accuracy of screw placement between the two groups. The deviation at the tip was 4.63 ± 2.72 mm in the experimental group and 7.38 ± 5.92 mm in the control group, showing a significant difference (*p* = 0.04). The deviation at the pedicle was 6.75 ± 3.06 mm in the experimental group and 10.15 ± 1.32 mm in the control group, with a statistically significant difference (*p* = 0.03). At the sacroiliac joint, the experimental group showed a deviation of 5.79 ± 3.38 mm, whereas the control group showed a deviation of 8.80 ± 5.78 mm, with a significant difference between the two groups (*p* = 0.03) **(**Table 1**)**.

### 3.6 Complications

No iatrogenic injuries related to the lumbosacral plexus, iliac arteries, or veins, as well as no postoperative surgical site infections, delayed fracture healing, or non-union of fractures, were observed in any of the patients.

## 4 Discussion

Unstable pelvic ring injuries usually refer to fractures of the pelvic ring accompanied by loss of the integrity of the pelvic ligaments. When compared with non-surgical treatment, surgical treatment with rigid internal fixation has significantly better radiological outcomes (reduction quality and deformity healing rate). From a functional perspective, surgical treatment has shown to significantly improve patients' functional recovery compared to non-surgical groups (14). Surgical treatment includes open surgery and minimally invasive surgery. The disadvantage of traditional open fixation is the need for extensive exposure of the deep pelvic structures, leading to guiding system currently longer surgery times, greater trauma, more blood loss, and potential damage to pelvic nerves and blood vessels. Additionally, the repeated bending of steel plates increases the risk of plate fractures (7).

For minimally invasive surgery, percutaneous screw fixation is a widely recognized technique for stabilizing pelvic ring injuries. It has significant advantages, including enhanced mechanical stability, minimal invasiveness, reduced blood loss, and lower infection rates (15), all of which contribute to faster postoperative recovery (16).

The application of E3D-assisted design combined with a personalized 3D printed guiding system, using bony landmarks as references and a rigid internal fixation device for metal insertion, ensures that the design of the guiding system is not influenced by skin morphology. This significantly reduces the deviation caused by the guiding plate, positioning needles, and skin deformation. Needle track infection is a common complication of external fixators (10, 17). Prior to applying the E3D-assisted design combined with the personalized 3D printed guiding system, positioning needles were inserted into the bilateral anterior superior iliac spines (two needles on the affected side and one on the unaffected side), increasing the potential for preoperative infection risks. To reduce this risk, preoperative needle track care was enhanced and efforts were made to minimize the preoperative preparation time. Preoperative planning and simulation of the surgery in the E3D software allowed for effective observation of the relationship between screws and vertebrae, avoiding damage to blood vessels and nerves. The average surgical time for the experimental group was 53.05 ± 19.04 min, which was shorter than the control group at 81.05 ± 24.68 min (*p* < 0.001). The experimental group placed 25 screws, while the control group placed 23 screws. The average fluoroscopy count in the experimental group was 48.60 ± 7.93, compared to 54.24 ± 9.32 in the control group (*p* = 0.044). The use of E3D-assisted design combined with personalized 3D printed guiding systems optimized preoperative planning, reducing intraoperative adjustments to the screw channel, which shortened the surgical time and reduced fluoroscopy frequency. However, conventional fluoroscopy is still needed preoperatively to determine the optimal pelvic entry, exit, and lateral views, providing a reference for intraoperative pelvic repositioning adjustments, followed by verification of the screw insertion position at the end of the surgery. The average blood loss during all screw placements was 27.54 mL, and the incisions were typically controlled to 1 cm, with good wound acceptance postoperatively. This guiding system, reverse-engineered from models simulated using the E3D software, ensures successful screw placement while verifying the accuracy of pelvic fracture reduction.

While previous studies have demonstrated the effectiveness of 3D-printed templates and navigation-assisted systems in improving screw placement accuracy, most approaches are based on CT reconstruction and rely on skin-surface-referenced guide plates. These methods may be affected by soft tissue deformation and often require costly navigation equipment. In contrast, the present study differs in several key aspects. First, E3D software was used as a comprehensive digital tool that integrates fracture visualization, surgical simulation, and screw channel planning, rather than serving solely as a reconstruction tool. Second, the guiding system was designed using a bone-anchored fixation strategy, in which positioning pins inserted into anatomical landmarks provide a rigid reference framework, thereby reducing deviations associated with soft tissue movement. It should be noted that while bone-referenced guiding concepts have been reported in previous studies, their application in pelvic fracture fixation remains limited. In this study, we further refine this concept by combining it with E3D-assisted planning and personalized guide design, improving both stability and reproducibility. Third, screw placement accuracy was assessed using a quantitative multi-point deviation method based on postoperative E3D reconstruction, which allows for a more detailed and standardized evaluation compared with conventional qualitative or single-point assessments. Taken together, these features represent a methodological refinement rather than a completely novel concept, providing a more integrated, practical, and potentially cost-effective approach for improving the precision of minimally invasive pelvic fracture surgery.

The E3D-assisted design combined with a personalized 3D printed has the following limitations: First, the process from design to application requires at least 3 days of preparation, which includes inserting positioning pins, importing CT data into software, planning the screw channel, reverse-engineering the guiding system, performing 3D printing, and finally sterilizing the template for use. This extends the surgical preparation time and affects the optimal timing for fracture reduction. In our study, the creation of the E3D-assisted design combined with a personalized 3D printed guiding system delayed the surgical timing. To eliminate the impact of this factor on the experiment, all surgeries in both groups were completed within two weeks of the injury. Additionally, after installing the 3D printed guiding system, there is only a single screw insertion channel, and it is not possible to fine-tune the screw channel through the 3D printed guiding system. The channel can only be adjusted using a Kirschner wire as a reference for the screw path. For this reason, we plan to design an adjustable channel in future 3D printed guiding systems to allow for finer adjustments to the screw path.

**Figure 3:**
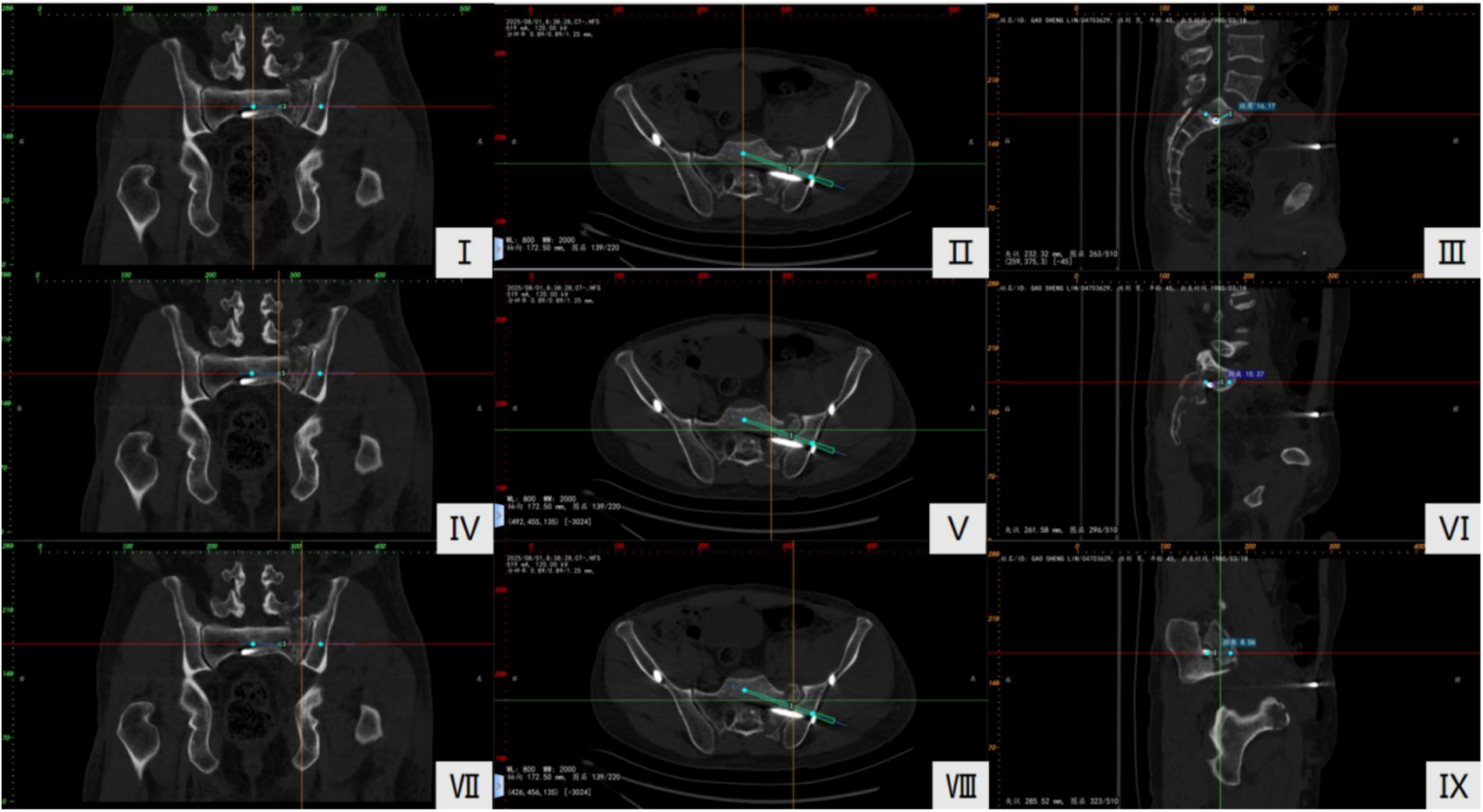
Measurement method of screw placement accuracy. (**I, IV, VII**) Select the height of the vertebra at 1/2 in the coronal view. (**II, V, VIII**) Plan the ideal screw on the cross-section and select measurement points at the tip, sacral vertebral arch, and sacroiliac joint. (**III, VI, IX**) Measure the deviation between the ideal and actual screw in the sagittal view. The red lines in the images represent the position of the cross-section in the coronal and sagittal planes, the yellow lines represent the position of the sagittal line in the coronal and cross-sections, and the green lines represent the position of the coronal view in the cross-section and sagittal planes.

Takao et al. (18) escribed a method to evaluate the deviation distance between the planned and actual IS screw, measured at the tip of the screw, the nerve root tunnel zone and entry point, on sagittal view CT images. Using 3D CT fluoroscopy matching navigation, they reported mean deviations of 2.2 ± 0.8 mm (tip), 1.8 ± 0.7 mm (nerve root tunnel) and 2.5 ± 1.8 mm (entry point). Takeba et al. (19) used a similar method with an O-arm navigation system, reporting an average deviation of 1.3 ± 0.6 mm for the screw tip position. Based on Takao et al.'s research, we set the ideal screw channel and compared it with the actual screw channel. Innovatively, we applied the E3D software to 1:1 restore the post-operative pelvic CT, using E3D software to achieve cross-connections between the pelvic CT coronal, sagittal, and axial views. This helped locate the screw placement height in the coronal view (Figures 3Ⅰ,Ⅳ,Ⅶ), selecting the vertebral height at 1/2 in the coronal view and creating an ideal screw channel in the axial plane, with the ideal screw placed at the center. Measurement points were selected at the tip (Figure 3Ⅱ), sacral pedicle (Figure 3Ⅴ), and sacroiliac joint (Figure Ⅷ) in the axial view, while cross-linking generated the images of the tip (Figure 3Ⅲ), sacral pedicle (Figure 3Ⅵ), and sacroiliac joint (Figure 3Ⅸ) in the sagittal view. The deviation between the actual screw and the ideal screw was measured at the tip, sacral pedicle, and sacroiliac joint in the sagittal view. The smaller the deviation, the closer the actual screw position is to the ideal screw position, thus proving higher screw placement accuracy.

This study demonstrates high clinical relevance, as improving screw placement accuracy while reducing operative time and radiation exposure remains a key objective in minimally invasive pelvic fixation. A notable innovation of this study is the use of a rigid, bone-anchored guiding system, which differs from traditional skin-based templates by minimizing the impact of soft tissue deformation and thereby enhancing positional accuracy. Furthermore, screw placement accuracy was evaluated using a reproducible and quantitative method based on postoperative CT reconstruction in E3D software, with measurements obtained at three critical anatomical points. The inclusion of clinically meaningful endpoints, including operative time, fluoroscopy frequency, blood loss, and functional outcomes, strengthens the translational value of our findings. Importantly, compared with navigation and robotic systems, the combination of E3D-assisted planning and 3D printing may represent a more cost-effective and accessible solution, particularly for primary healthcare settings. However, this study is limited by its retrospective design and relatively small sample size. Although no iatrogenic complications or infections were observed, further large-scale, prospective, randomized studies are required to confirm the safety and generalizability of this approach.

Currently, various auxiliary tools have been used to improve the accuracy and efficiency of guide pin insertion, including C-arm fluoroscopy-guided screw placement, CT-guided screw placement, robotic-assisted screw placement, and 3D-printed assistive templates. A series of studies have been conducted both domestically and internationally focusing on percutaneous sacroiliac screw technology. Jui-Ping Chen et al. (20) conducted an efficacy analysis using C-arm for percutaneous iliosacral screw fixation, concluding that C-arm guidance is a reliable and safe auxiliary screw placement technique. Using C-arm for percutaneous iliosacral screw fixation typically involves guiding the insertion through the inlet, outlet, and lateral fluoroscopic views, but cortical penetration remains a common issue with iliosacral screw implantation (21, 22). This method, like open surgery, also carries the risk of damage to pelvic nerves and blood vessels during the procedure. Additional fluoroscopic views are often required to further improve accuracy. Ozmeric et al. (23) suggested using two different inlet views to evaluate the anterior and posterior boundaries of the sacrum. Kim et al. (21) even recommended using two inlet views (at 25° and 55° angles to the vertical plane) and three outlet views (at 25°, 35°, and 55° angles to the vertical plane) to avoid misjudging local anatomical structures. However, any adjustment to the guide pin position in any view must be re-verified across all fluoroscopic views. Thus, improving the accuracy of guide pin insertion using traditional methods inevitably increases both operation time and radiation exposure because the process from planning the screw path to insertion requires time. This limits the application of traditional C-arm-guided sacroiliac screw insertion techniques in clinical practice. With the development of 3D printing technology, it has been widely applied in orthopedics. Bin Chen et al. (24) developed a 3D reconstruction and reverse engineering template based on CT scans and found that compared to the traditional sacroiliac screw group, the CT-based personalized template resulted in more accurate screw placement, less radiation exposure, and shorter surgical times. Kaifang Chen et al. (25) developed a 3D-printed external template dependent on Kirschner wire positioning technology for minimally invasive treatment of unstable pelvic fractures, confirming that the technique with a 3D-printed external template is a safe and effective method for placing sacroiliac screws in unstable pelvic fractures, minimizing surgery time and radiation exposure. Yizhou Wan et al. compared the efficiency of percutaneous sacroiliac screw placement using 3D templates and computer-assisted navigation, concluding that both techniques increased surgical efficiency, but the template group had shorter operation times, although preoperative preparation time was longer. Fan Yang et al. (26) described using a new patient-specific external template to guide sacroiliac screw insertion and compared this technique with traditional fluoroscopy-guided methods, concluding that the external template provides an accurate and safe navigation tool for percutaneous sacroiliac screw placement, reducing surgery time and radiation exposure. Feng Liu et al. (27) showed that the use of 3D-printed templates for sacroiliac screw fixation in stable pelvic ring fractures is both feasible and safe, reducing fluoroscopy time, screw placement time, and intraoperative blood loss, while achieving good postoperative recovery. The 3D-printed templates, which guide surgeons in screw insertion during surgery, have been effective, but they lack the ability to verify pelvic fracture reduction. We hope that the combination of E3D-assisted design and personalized 3D-printed guiding systems will assist surgeons in improving the efficiency and accuracy of sacroiliac screw placement, and help introduce this minimally invasive surgery in basic-level hospitals without access to robotics and CT navigation.

This study has several important limitations. First, its retrospective design and lack of randomization introduce potential selection bias and limit the ability to establish causal relationships. Although we included all consecutive patients who met the eligibility criteria during the study period, selection bias cannot be completely excluded. Second, the sample size was relatively small (*n* = 41), and no formal power calculation was performed, which may limit statistical power and the generalizability of the findings. Third, due to the nature of the intervention, blinding of surgeons and patients was not feasible, and outcome assessors were not formally blinded, which may introduce performance and detection bias. In addition, inter-rater reliability for radiological measurements and reduction quality assessment was not evaluated. Fourth, the description of the control group has now been clarified; screw placement relied on conventional fluoroscopy-guided intraoperative planning without the use of dedicated preoperative digital simulation software. Fifth, intraoperative blood loss was estimated using standard clinical methods based on suction volume and gauze weighing; however, this approach may be subject to measurement variability. Sixth, surgeries were performed by experienced surgeons within the same team rather than a single operator, and a potential learning curve effect associated with the use of the 3D-printed guiding system cannot be excluded. Finally, the follow-up period was limited to six months, which is insufficient to assess long-term functional outcomes, implant-related complications, and late screw loosening. Future prospective, multicenter studies with larger sample sizes and longer follow-up durations are warranted to validate and extend these findings. In addition, although screw placement accuracy measurements were performed independently by two observers, formal intra- and inter-observer reliability analyses were not conducted, which may introduce measurement bias.

## 5 Conclusion

The study found that when comparing the application of E3D-assisted design combined with personalized 3D printed guiding systems to traditional fluoroscopy-guided minimally invasive screw placement techniques, the use of E3D-assisted design and 3D printed systems enhanced surgical efficiency, screw placement accuracy, and safety.

## Data Availability

The original contributions presented in the study are included in the article/supplementary material, further inquiries can be directed to the corresponding author.
